# Particulate Matter Exposure across Latino Ethnicities

**DOI:** 10.3390/ijerph18105186

**Published:** 2021-05-13

**Authors:** Kerry Ard, Dax Fisher-Garibay, Daphney Bonner

**Affiliations:** School of Environment and Natural Resources, Ohio State University, Columbus, OH 43210, USA; fisher-garibay.1@osu.edu (D.F.-G.); bonner.190@osu.edu (D.B.)

**Keywords:** particulate matter, industrial, air pollution, Hispanic, chemicals

## Abstract

The Hispanic/Latino health paradox is the well-known health advantage seen across the Hispanic/Latino racial category in the US. However, this racial category collapses several distinct ethnic groups with varying spatial distributions. Certain populations, such as Dominicans and Cubans, are concentrated in specific areas, compared to more dispersed groups such as Mexicans. Historical peculiarities have brought these populations into contact with specific types of environmental exposures. This paper takes a first step towards unraveling these diverse exposure profiles by estimating how exposure to particulate matter varies across demographic groups and narrows down which types of industries and chemicals are contributing the most to air toxins. Exposure to particulate matter is estimated for 72,271 census tracts in the continental US to evaluate how these exposures correlate with the proportion of the population classified within the four largest groups that make up the Hispanic population in the US: Mexican, Puerto Rican, Cuban, and Dominican. Using linear mixed models, with the state nested within US Environmental Protection Agency regulatory region, and controls for population density, we find that the Dominican population is significantly less exposed to PM_2.5_ and PM_10_ compared to non-Hispanic Whites. Moreover, those tracts with a higher proportion of Cuban residents are significantly less exposed to PM_2.5_. However, those tracts with a higher proportion of foreign-born, Mexicans, and Puerto Ricans had significantly higher levels of exposure to all sizes of particulate matter. We discuss the need to consider the chemical components of these particles to better understand the risk of exposure to air pollution.

## 1. Introduction

The Hispanic health paradox is the well-documented health advantage held by Hispanic Americans (herein referred to as Latino) compared to non-Hispanic white Americans [1]. Previous work has shown that Latino individuals have better self-rated health, lower morbidity, and mortality than their socioeconomic status would predict [2]. However, this health advantage is not uniform across all Hispanic groups and varies by the health outcome being measured [1]. For example, Dominicans have lower asthma prevalence than Puerto Ricans (5.3% and 13.2%, respectively) [3]; favorable mortality outcomes apply most strikingly to Mexican Americans [2], yet infant mortality is lowest amongst Cubans [4]. A study examining 17 years of data from the National Health Interview Survey, found Puerto Ricans were the most likely to have reported a chronic illness, and have a lower self-rated health, compared to Mexican, Cuban, and Dominican respondents [5]. Scholars have attempted to explain the diversity of these health outcomes across the Latino subgroups by examining differences in access and use of health care [6], acculturation [7], health behaviors [8], and skin color [9]. This work has provided a great deal of understanding of the mechanisms underlying these patterns. However, exposure to environmental toxins likely varies along ethnic lines and there has yet to be an examination of these relationships at a national level.

Different land uses, such as industrial activity or transportation lines, cluster together in space due to zoning, as well as the ability to utilize similar resources (e.g., landscape features, employment base, tax exemptions). Therefore, the types of toxins resulting from these activities will vary in space as well. Moreover, we can expect that because different ethnic groups have differential representation in these activities, such as the Mexican population in the agricultural industry [10], and historically distinct settlement areas, such as Dominicans in the Northeast, we can expect the average health risk from these exposures to also vary by ethnic group. The handful of research investigating these patterns supports this argument. For example, in a study of Miami metro-area residents, the cancer risk from on-road sources was higher for Cuban neighborhoods compared to Mexican areas [11]. Another study of air-pollution-associated hospitalizations in El Paso, Texas, found that, compared to whites, Hispanics were at a lower risk for NO_2_ associated admissions from 2005 to 2010, yet at a greater risk for PM_2.5_ admissions. Variations in exposure to particulate matter are a useful starting place to better understand these ethnic patterns of exposure as both long-term and short-term exposure to particulate matter have been associated with all-cause and cause-specific mortality [12].

PM_2.5_ and PM_10_ measure different sizes of inhalable particles, or particulate matter, with PM_2.5_ referring to particles that are 2.5 micrometers, and PM_10_ referring to particles that are 10 micrometers, or smaller. Both PM_2.5_ and PM_10_ can occur from a variety of sources depending on the geography of where they are being measured. For example, in a study done in Berlin, Germany in 1990 and 1998, it was found that the urban background of PM_10_ consisted of soil, ash, inorganic secondary aerosols, and carbonaceous material [13]. However, on a busy street, the PM_10_ pollution was 40% higher than these background levels, with a little over half of that increase due to exhaust emissions and tire abrasion [13]. The World Health Organization notes the importance of considering the geographic distribution of possible sources, noting the most common components of PM_10_ are from industrial activities as well as mechanical processes and road dust suspension, while PM_2.5_ is noted to come primarily from combustion sources [14]. Due to their smaller size, PM_2.5_ can go deeper in the lungs than PM_10_, and there is increasing evidence that the smaller particulate size the stronger the health insult on the cardiovascular system [15,16]. Yet, even coarser particulate matter, such as PM_10_, has been associated with negative health outcomes. A metanalysis of over a million participants found that both long-term and short-term exposure to PM_10_ was associated with a risk of depression and suicide [17].

While the size of the particle tells us how deeply it is likely to be inhaled into the lungs, it tells us nothing about its chemical components. Researchers have raised concerns about the thousands of chemicals that are on the market without adequate testing [18]. With the National Academies of Sciences noting, prior to the passage of the 2016 Frank R. Lautenberg Chemical Safety Act, the US EPA, “allowed approximately 82,000 potentially unsafe chemicals to remain in the US [19].” Moreover, scholars worry that traditional approaches to risk assessment will never allow us to catch up to evaluate related health risks, and instead advocate moving towards predictive models [20]. The vast number of chemicals that individuals are exposed to makes it difficult for public health scholars to sufficiently measure one’s exposome—the totality of health insults over a complete lifetime [21]. Exposome models are developed to understand how one’s biography becomes their biology [21]. To achieve this understanding also requires consideration of the geography of one’s life and the chemical risks within these spaces.

To gain a more complete understanding of how environmental risks cluster in space we can first look towards industrial air toxins. Industries that use similar types of manufacturing processes and methods often cluster together in space [22]. Specific geographic locations likely provide the same benefits to facilities within the same industry, such as necessary infrastructure (e.g., waterways and highways) and access to markets and labor that companies need to effectively do business. This process creates a “zone of industry” in which we would expect chemical combinations from the predominant industry to be more prevalent in the environment. For example, steel industries use manganese and chromium to enhance durability and corrosion resistance. Thus, we would expect these two chemicals to be found together in the environment more often and have a higher chance of interacting with one another. Those interested in understanding environmental health risks need to work towards disentangling how industries, chemicals, and demographic groups cluster in space, and how these patterns might influence susceptibility to different environmental hazards. To begin this process, we look towards where different demographic groups within the Latino population have historically settled in the US.

### Spatial Patterns of the Latino Population

The subpopulations that make up the Hispanic racial category in the US have been relatively geographically distinct [23]. Mexicans are, and historically have been, the largest Hispanic subgroup living in the US, making up roughly 11 percent of the total population in 2019 [24,25]. Today, most Mexican Americans continue to reside largely in southwest states often taking advantage of employment opportunities in farming, ranching, and mining [19]. In 2014, roughly a quarter of the US agriculture industry’s employment base were Latino [26]. This would make this group particularly exposed to particulate-matter that arises from dust particles and pesticide residue, as well as food and wholesale industrial pollution. After testing the blood serum of 26 Mexican migrant farmworkers in Texas, scholars found that participants who had worked for longer in the agriculture industry had higher levels of organochlorine pesticides in their blood [27]. Nevertheless, newer waves of Mexican Americans are beginning to migrate to secondary cities in Colorado, Florida, Georgia, Indiana, and Illinois, largely due to a switch from seasonal work to more permanent, year-round jobs in service industries, and construction, where the Mexican immigrants now make up over one-third of workers, putting them at a greater risk of exposure to diesel exhaust [28].

Puerto Ricans are the second-largest Latino group in the US, making up about 1.7 percent of the population in 2019 [18]. Though US citizens, islanders did not travel to the continental US in large numbers until the end of World War II where they settled largely in New York, and currently make up 5.4 percent of the state population [18]. They have also settled in other East Coast and midwestern states such as Connecticut, where they make up 8.2 percent of the state population, Massachusetts (4.8% of the population), New Jersey (5.4% of the population), New York (5.6% of the population), and Florida (3.4% of the population) [18]. Due to the economic poverty on the island, those migrating from Puerto Rico likely have lower incomes compared to the mainland neighborhoods where they settle. A recent study of allostatic load amongst the Latino ethnic groups showed that Puerto Rican men are at increased risk of biological dysregulation due to chronic stress [29]. Unlike the Mexican population, only a very small percentage of the mainland Puerto Rican population is employed in the agriculture industry, instead, over a quarter are in the management, professional, technical, sales, and service industries [30]. Such employment positions would likely make this group more exposed to emissions from transportation exhaust due to the more urbanized locations of these industries.

Cubans and Dominicans are the third and fourth-largest Hispanic subgroups and the most geographically isolated. For example, although the Cuban population composes 1.7 percent of the US population, they make up 7.3 percent of Florida’s population [18]. Prior to 1959, tens of thousands of Cubans immigrated freely between Florida and Cuba for work [31]. In the early 1960s when Castro took over the island nation, more than 200,000, mostly wealthy, white, professional immigrants fled to the US. Soon after this, the US passed the Cuban American Adjustment Act of 1966 which allowed any Cuban who lived in the US for more than a year the ability to become a permanent resident; this led to more than 300,000 Cubans immigrating to the US [24]. Currently, Cubans are highly represented in the suburbs [32]. Overall, Cuban Americans typically have higher economic, social, and political power than other Latino subgroups [33] and higher levels of neighborhood social capital [34]. A study of over 15,000 immigrants found that Cubans have the lowest self-reported depression amongst all the Latino ethnic groups [35], supporting prior work with similar findings [36].

Dominicans are as spatially isolated as the Cuban subgroup, with 60% of the entire Dominican population in the US residing in NYC, consisting of 4.4 percent of New York state’s population [18,19]. Dominicans also make up 5% of the small, and largely urban state, Rhode Island. Since the 1970s, Dominicans are highly concentrated within New York City in Washington Heights and Corona neighborhoods; despite having relatively higher rates of poverty, Dominicans have been less likely than other Hispanic subgroups to be living in public housing due to more recent immigrant groups being largely shut out of this program due to long waitlists [23]. Today, Dominicans are most “likely to be employed in service occupations or production, transportation, and material moving occupations [19].” A recent study of the health data of Dominicans located in New York City found that the prevalence of obesity was lower for Dominicans compared to Mexicans and Puerto Ricans. In a national-level study of roughly 7500 US adults, Dominicans and Cubans had the lowest prevalence of diabetes (19.3%) compared to Mexicans and Puerto Ricans (24.6% and 20.5%, respectively) [37].

Latino subgroups have diverse spatial patterns that vary across gradients of urbanization, labor, and other economic factors. Understanding how these diverse settlement histories relate to environmental risk is currently under-explored. The following analyses contribute to this literature by examining how the ethnicities that make up the Latino population are related to particulate matter exposure and the most prevalent industrial air toxins. We focus on the four largest ethnic subgroups of the Hispanic racial classification in the US census: Mexican, Puerto Rican, Cuban, and Dominican.

## 2. Materials and Methods

Demographic data were obtained for all census tracts within the continental US from the American Community Survey (ACS) 2015 5-Year estimates via Social Explorer. Population data were obtained at the tract level for non-Hispanic whites and blacks (herein referred to as whites and African Americans). For those individuals who classified themselves as: “Hispanic”, “Latino”, or “Spanish”, information was collected on whether they considered themselves as “white-Hispanic” or “black-Hispanic”. These data were utilized in the analyses below. In addition, the ACS 2015 also gathered ethnic origin information for those census respondents that classified themselves as “Hispanic”, “Latino”, or “Spanish”. Individuals who chose that option could also record which ethnic group they associated themselves with. The following analyses were based on those whose ethnic origin was from one of the largest four groups: Cuban, Dominican, Mexican, and Puerto Rican. Data were also obtained on the proportion of the tract population that was foreign-born and unemployment by race (i.e., white, African American, and Latino). Twenty-four tracts were dropped for not having demographic data and 519 for not having estimated pollution exposure estimates for a total of 72,562 census tracts.

Census tract-level pollution estimates for particulate matter the size of 2.5 micrograms (PM_2.5_) and 10 micrograms (PM_10_) micrograms per cubic meter were obtained from the Center for Air, Climate, and Energy Solutions database based out of Carnegie Mellon University for the most recently available year, 2015, for the continental US. Estimates of exposure for outdoor concentrations of PM_2.5_ and PM_10_ are based on models using publicly available data from the US EPA, land use, and satellite-derived estimates of exposure [38]. While the size of particulate matter tells us the propensity for the particulate to be inhaled more deeply into the lung, it does not tell us anything about the chemical components of these particles. To better elucidate what types of air toxins the different demographic groups experience, we begin our analysis with an examination of the types of chemicals and industries that contribute the most to the health risk across US Environmental Protection Agency’s (USEPA) enforcement regions. These data were obtained from the USEPA Toxic Release Inventory program. The EPA provides these data in a program called EasyRSEI [39], which allows users to download information about pollution emissions by industry categorized by their primary 3-digit 2012 North American Industry Classification System (NAICS) code. These data were summarized across the US EPA Region and the health risk estimated for each chemical type regulated was noted [40]. The EPA region was chosen rather than the census region because it is at this level of analysis that environmental policies related to the outcome variable are decided [41]. In addition, the locations of the 57,120 TRI facilities in the continental US were overlaid with census tracts and categorized by their primary three-digit NAICS code, of these, 7445 were located in census tracts that the ACS did not have information for and were dropped. All data utilized are publicly available.

## 3. Results

**Figure 1:**
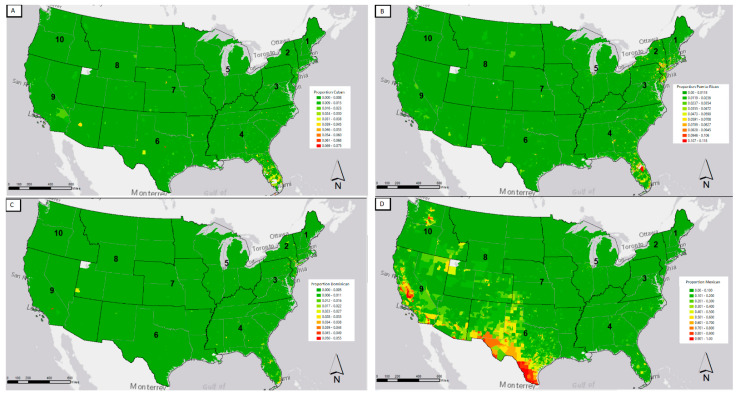
Map of proportion of census tract for four Latino ethnic groups, broken down by decile; (**A**) Proportion of Cuban population; (**B**) Proportion of Puerto Rican population; (**C**) Proportion of Dominican population; (**D**) Proportion of Mexican population.

This paper aims to shine more light on the variation in exposure to air pollution across the US and how these risks relate to the Latino population. We can first look at Figure 1 to see the geographic distribution of the four largest Latino ethnic groups. The proportion of a census tract population that fell within the four demographic groups examined was broken down into deciles within each group, with those census tracts falling in the highest decile being denoted as red, and the lowest as green. Figure 1 shows the average percentile of each demographic group broken down by the EPA region. We can see that the Mexican population is distributed largely in the Southwest, EPA regions 6, 9, and 10. While the Cuban population is largely in Florida, region 4, with Puerto Ricans in region 4, as well as regions 1 and 2, with Dominicans located mainly in region 2. These population distributions are largely as expected. We next turn to how pollution exposure varies across these landscapes.

**Figure 2:**
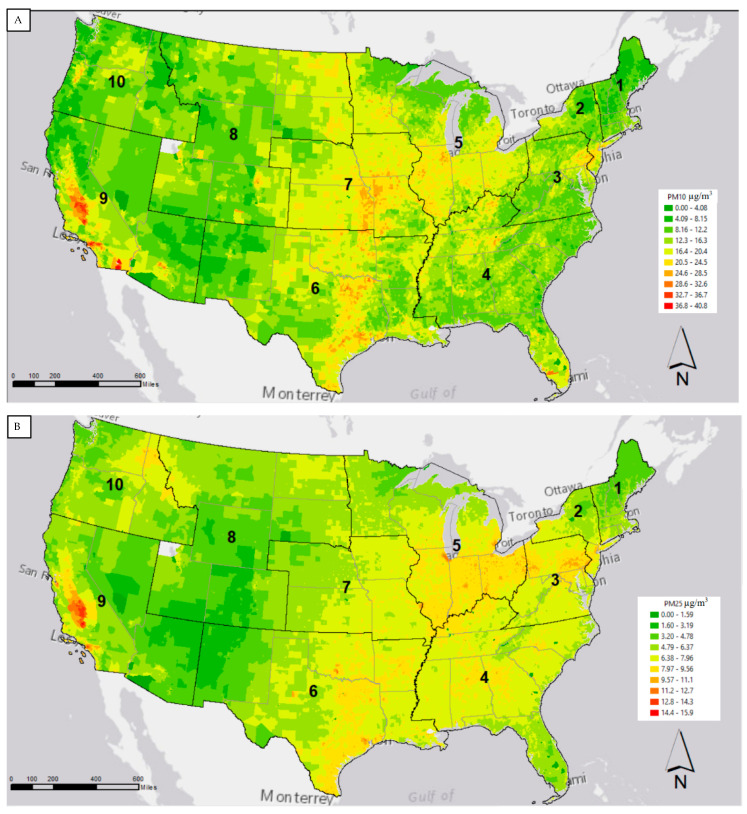
Census tract-level estimates of (**A**) PM_10_ and (**B**) PM_2.5,_ measured in micrograms per cubic meter, for the year 2015 color-coded by deciles.

We can look at Figure 2 to see a map of the distribution of PM_2.5_ and PM_10_. Recall, the census tract level estimates of particulate matter, measured in micrograms per cubic meter, were utilized. These estimates were then broken down into deciles within each particle size, ranging from 0.0 to 15.9 micrograms per cubic meter for PM_2.5_, and 0.0 to 40.8 micrograms per cubic meter for PM_10_. We see hotspots in the central valley of California for both sizes of particulate matter. The central US and Texas have high levels of PM_10_, while the higher deciles of PM_2.5_ are seen in the Midwest, as well as parts of Texas and the south. To try to understand possible source chemicals, we moved on to examining how industrial types and chemicals vary across this landscape.

To better understand the spatial distribution of EPA-regulated industrial facilities by their primary three-digit North American Industry Classification System code, we can look to Table 1. There were 420 different types of industry represented in these data. Table 1 lays out the top ten for each region, demonstrating that there are eight categories that fall into the top three types of facilities in each region. The chemical manufacturing industry makes up the largest portion of facilities in this dataset, followed by fabricated metal, food industry, computer and electronic manufacturing, primary metal manufacturing, plastics and rubber, wood products, and transportation equipment manufacturing. Regions where the Mexican population is most highly represented are distinct in their high number of food and wood product manufacturing industries. Region 4, where the Cuban population is most highly represented is unique in its density of plastics and rubber industry facilities. Whereas computer and electronic manufacturing is most likely to take place in regions 1 and 2, where the Puerto Rican and Dominican populations cluster.

Table 2 displays the Pearson correlations between the proportion of census tract residents in one of the four demographic groups examined, and the density of facilities in that tract that fall into one of the NAICS codes that made up the largest number of industrial facilities. These relationships demonstrate that no matter the industry type, the proportion of the white population is negatively related to the density of facility type. In general, the relationships between density of industrial facility, no matter the type, was positively related to the proportion of those within the Latino groups examined, with fabricated metal manufacturing being the most strongly related across all groups, and Puerto Ricans having the largest correlations across all industry types.

Table 3 breaks down the air toxins that contribute the most to the estimated health risk of the EPA region. These data demonstrate many similarities in exposure risk across regions 2 and 4, where the Puerto Rican/Dominican and Cuban populations are more likely to reside, respectively. For example, chromium, and its compounds, contribute the greatest to the overall health risk across regions and has been related to asthma as well as noncancer respiratory deaths [42]. Cobalt, and its compounds, are high in these regions as well, and have been related to lung cancer and systemic tumors in rats [43]. Ethylene oxide is also a major contributor to risk and has been linked to lymphoid and breast cancers [44]. However, region 4 is comparatively distinct in its high exposure to arsenic compounds, which is associated with lung cancer [45], while region 2 is comparatively distinct in its higher exposure to nickel air toxins, associated with respiratory tract cancers [46]. EPA region 6, where the Mexican populations are likely to reside, has the most diverse chemical profile with chemicals like 1,3-butadiene, which is associated with leukemia [47], a similar chemical chloroprene which is associated with multiple cancers in animal studies [48].

Table 4 lays out the result of mixed models predicting tract PM_10_ exposure. Model 1 limits the predictor variables to the common racial categories in the US census, non-Hispanic whites, African Americans, and Hispanics. Model 1 demonstrates a one proportion increase of Hispanic residents in a tract was associated with an increase of almost six micrograms per cubic meter of estimated particulate matter. However, the following models break this racial category down into its component parts where we see more variation. For example, model 2 breaks down the Hispanic racial group into Hispanic whites and Hispanic Blacks, with Hispanic whites being strongly related to higher rates of exposure compared to Hispanic blacks. Further analysis shows that those ethnic groups that largely make up each of these categories were Mexicans and Dominicans, respectively. Even when including a proportion of foreign-born people in this model (model 3), these patterns remain.

The remaining models work to disentangle the different subgroups that make up the broader Hispanic population. Model 4 demonstrates that, on average, the Cuban population is most strongly exposed to PM_10_ while the Dominican population is protected from PM_10_ compared to the white reference group. Including the proportion of foreign-born (model 5), most of the patterns remain with some of the variation associated with the Cuban population now being explained by the population of foreign-born. Consistently, the proportion of African Americans was associated with a significant increase in exposure to particulate matter in these models, this relationship varied relatively little across models. Interestingly the economic variables were negatively related to exposure, possibly due to increases in particulate matter associated with economic activity in these areas. However, this model had the lowest BIC suggesting the best fit compared to the other models. Notably, the relationship between estimated PM_10_ exposure was not significantly related to the proportion of unemployed whites in that census tract, unlike the other groups examined.

Table 5 examines if these relationships change when predicting PM_2.5_. In fact, we see the correlations are smaller, possibly because this particle size can be transported farther by wind patterns due to its small size, and therefore, local characteristics are less predictive than if we were measuring pollutants closer to their source, such as PM_10_. Nevertheless, it provides an interesting comparison. Overall, we see similar patterns in Table 5 that we saw in Table 4. Such as the relatively consistent patterns between the proportion of African American and PM_2.5_ exposure, with a higher proportion of African American associated with roughly one and a half times greater amounts of PM_2.5_. compared to whites. Again, Hispanic whites were related to greater exposure, and Hispanic blacks were related to significantly less exposure to PM_2.5_, compared to non-Hispanic whites. In model 5, we observe the biggest difference between the PM_2.5_ and the PM_10_ results. While Table 4 showed that Cubans were significantly more exposed to PM_10_ compared to whites, they are shown in Table 5 to be significantly less exposed to PM_2.5_ (model 5). This relationship held when including the proportion of those who were unemployed by racial group in model 6. Again, we see when controlling for the percentage of unemployed that we have the best fitting model, as determined by the lowest BIC.

## 4. Discussion

Having a better understanding of how types of environmental risk vary across space can help health scholars develop more accurate health risk models. The analyses presented here provide patterns of exposure to environmental toxins which might be helpful as public health scholars begin to disentangle the various health insults which influence one’s exposome, an individual’s lifetime exposure to health risk. While the analyses presented here only offer us a first step, they do demonstrate that there is a distinct variation in environmental risk by demographic group and location that should be considered in future work. For example, in our examination of how industry type and air toxin risk varied by EPA region, we found that, for those industries regulated under the Toxic Release Inventory program, regions where the Mexican population is most highly represented have more food and wood product manufacturing industries. Moreover, these regions have a higher risk of exposure to a chemical related to leukemia compared to other EPA regions. On the other hand, the region where Cuban populations are most located has a higher density of plastics and rubber industry facilities, as well as a relatively greater risk from chemicals linked to lung cancer. Finally, areas with the largest proportion of the Puerto Rican and Dominican population in the US are located around more computer and electronic manufacturing facilities and associated with chemicals that have been related to respiratory tract cancers.

While this study cannot provide us information on actual exposures of individuals to air toxins, it can help to move our thinking towards considering how environmental risk covaries with demographics in the US. Health practitioners are increasingly recognizing the importance of one’s social and physical environment in their patients’ health outcomes. Concepts such as the social determinants of health [49], and the exposome [50] require new thinking and operationalization of riskscapes. Scholars working in this area have derived algorithms to parse out relationships between socio-demographic variables and highlight those which are the most meaningful [51]. Such work needs to be done for air toxins. Research on the size of the particulate matter is important, however health researchers need to recognize that the possible chemical variation in the particle make-up could vary spatially.

In addition to the variation in the chemical makeup of particulate matter, exposure to particulate matter also varies spatially and in a way that interacts with demography. In this paper, we present results from linear mixed models that control for population density, and nesting of states with EPA region, that estimate the relationships between ethnicity and census tract exposure to PM_10_ and PM_2.5_. We look specifically at the four largest subgroups that make up the Latino population in the US. We find that while overall, the Hispanic population in the US is more exposed to air pollution, this exposure varies by subgroup. Specifically, we find that compared to whites at a national level, Dominicans and Cubans are less exposed to PM_2.5_ than whites, while Mexicans and Puerto Ricans are more exposed. Patterns were similar when estimating exposure to PM_10_, apart from the Cuban population being significantly more exposed to PM_10_. Looking at the maps of PM_10_ and PM_2.5_ we can see that PM_10_ is relatively higher in Florida, where the majority of Cubans live, than PM_2.5_, which are the smaller particles that disperse over a wider geography.

One of the most striking relationships in these analyses was the relatively strong, and negative, relationship between the proportion of Dominicans living in a tract and exposure to particulate matter. This relationship held even when including population density, measures of unemployment, and percent foreign-born. The strong negative relationship between Dominicans and particulate matter likely explains the similarly negative relationship between Hispanic blacks and exposure, as Dominicans make up the majority of this racial category. This is an interesting finding considering that black immigrants likely experience greater discrimination as they enter into a highly racialized structure in the US [52]. Nevertheless, research on the spatial distribution of these populations in the US from 1990 to 2010 shows the Dominican population is the most isolated of all the ethnicities, and importantly, the most spatially segregated from the ethnicity with the highest estimated exposure, the Mexicans [53].

Future work should expand upon this study and endeavor to overcome our limitations. The Center for Air, Climate, and Energy Solutions database based out of Carnegie Mellon University is a wonderful new resource for particulate matter estimates. Their methods have allowed for methodologically sound and easily accessible data on particulate matter. However, these data are limited in that they do not have the chemical makeup of the particles being inhaled. We encourage greater problematizing of the complexities of the components of particulate matter and its role in health outcomes. Such questions are important if we are to understand how and why exposure to particulate matter is related to other factors such as COVID-19 infection [54]. While this study was limited to the year 2015 for particulate matter exposure estimates, future work should examine how such patterns changed during quarantines across the US. Finally, linking these patterns to the health outcomes of individuals is an important step future work should attempt to take. Having a complete understanding of the complexity of environmental health insults on individuals across their lifetime could lead to the better targeting of health interventions.

## 5. Conclusions

In this paper, we consider the spatial distribution of the four largest subgroups of the Latino population: Mexican, Puerto Rican, Cuban, and Dominican, and estimate how these spatial distributions relate to pollution exposure. We find important variation across subgroups. For example, Dominicans and Cubans were observed to be significantly less exposed to PM_2.5_ compared to whites, Mexicans, and Puerto Ricans were significantly more exposed. In addition, we work to characterize the spatial distribution of the types of industry and air toxins that affect the US. Having a better understanding of the chemical components of particulate matter could help to better understand health risks from environmental toxins. These analyses help to take a first step towards incorporating estimates of how geographic histories relate to variation in exposure to toxins in our environment. Future work should investigate how exposure levels and chemical combinations affect demographic groups differently. Having a better understanding of how differential exposure to environmental toxins varies by demographic group could be the key to understanding certain public health patterns.

## Figures and Tables

**Table 1:** Location of USEPA regulated facilities organized by their primary three-digit North American industry classification system for each of the ten US EPA regions.

EPA Region 1		EPA Region 2		EPA Region 3		EPA Region 4		EPA Region 5	
**NAICS 3 Digit Code**	**%**	**NAICS 3 Digit Code**	**%**	**NAICS 3 Digit Code**	**%**	**NAICS 3 Digit Code**	**%**	**NAICS 3 Digit Code**	**%**
Fabricated Metal	18.36	Chemical	23.32	Chemical	14.18	Chemical	15.18	Fabricated Metal	18.46
Computer and Electronic	10.94	Fabricated Metal	13.17	Fabricated Metal	13.49	Fabricated Metal	9.89	Chemical	13.27
Chemical	10.75	Computer and Electronic	6.60	Primary Metal	8.25	Plastics and Rubber	7.84	Transportation Equipment	8.89
Miscellaneous Manufacturing	6.24	Primary Metal	6.12	Food	6.41	Transportation Equipment	7.63	Primary Metal	8.50
Primary Metal	5.93	Food	5.45	Plastics and Rubber	6.26	Food	6.73	Plastics and Rubber	7.78
Plastics and Rubber	5.47	Plastics and Rubber	4.48	Nonmetallic Mineral Product	5.73	Wood Product	5.85	Food	6.66
Elec. Equip., Appliance, Component	4.94	Merchant Wholesaler, Nondurable	4.03	Machinery	4.91	Primary Metal	4.90	Machinery	5.99
Machinery	4.91	Machinery	4.00	Computer and Electronic	4.91	Nonmetallic Mineral Product	4.77	Computer and Electronic	4.62
Paper	4.54	Nonmetallic Mineral Product	3.91	Transportation Equipment	4.25	Machinery	4.29	Nonmetallic Mineral Product	3.42
Nonmetallic Mineral Product	4.22	Paper	3.69	Wood Product	3.49	Textile Mills	3.97	Elec. Equip., Appliance, Component	3.08
**Total Region 1 TRI Facilities 3129**		**Total Region 2 TRI Facilities 3302**		**Total Region 3 TRI Facilities 4521**		**Total Region 4 TRI Facilities 9896**		**Total Region 5 TRI Facilities 12,590**	
**EPA Region 6**		**EPA Region 7**		**EPA Region 8**		**EPA Region 9**		**EPA Region 10**	
**NAICS 3 Digit Code**	**%**	**NAICS 3 Digit Code**	**%**	**NAICS 3 Digit Code**	**%**	**NAICS 3 Digit Code**	**%**	**NAICS 3 Digit Code**	**%**
Chemical	19.13	Food	16.47	Chemical	11.60	Chemical	14.57	Wood Product	12.72
Fabricated Metal	12.39	Chemical	14.80	Fabricated Metal	10.99	Computer and Electronic	14.04	Food	11.90
Food	8.06	Fabricated Metal	11.09	Food	9.64	Fabricated Metal	13.40	Chemical	10.65
Transportation Equipment	6.11	Transportation Equipment	7.61	Nonmetallic Mineral Product	9.19	Nonmetallic Mineral Product	6.55	Fabricated Metal	9.15
Plastics and Rubber	6.09	Machinery	7.22	Computer and Electronic	8.13	Food	6.18	Computer and Electronic	7.96
Merchant Wholesale, Nondurable	5.79	Plastics and Rubber	6.23	Machinery	4.97	Plastics and Rubber	5.47	Transportation Equipment	7.89
Machinery	5.50	Nonmetallic Mineral Product	5.51	Plastics and Rubber	4.82	Transportation Equipment	5.22	Nonmetallic Mineral Product	6.39
Primary Metal	5.14	Primary Metal	4.43	Transportation Equipment	4.52	Primary Metal	4.22	Primary Metal	5.45
Nonmetallic Mineral Product	4.81	Elec. Equip., Appliance, Component	2.95	Miscellaneous Manufacturing	4.29	Machinery	3.45	Plastics and Rubber	5.26
Computer and Electronic	4.41	Computer and Electronic	2.89	Petroleum and Coal Products	4.07	Merchant Wholesale, Nondurable	3.22	Machinery	3.57
**Total Region 6 TRI Facilities 5077**		**Total Region 7 TRI Facilities 3048**		**Total Region 8 TRI Facilities 1328**		**Total Region 9 TRI Facilities 5098**		**Total Region 10 TRI Facilities 1596**	

**Table 2:** Pearson correlations between census tract population of Latine groups, and the density of USEPA regulated facilities organized by their primary three-digit North American industry classification system.

		Census Tract Density of Industrial Facilities by Primary 3-Digit NAICS Code									
		Wholesale	Machinery	Primary Metal	Computer & Electronics	Mineral	Plastics & Rubber	Transport.	Food	Fab. Metal	Chemical
Proportion of Tract Population	Dominican	0.08	0.03	0.08	0.07	0.06	0.03	0.05	0.05	**0.11**	**0.20**
	Cuban	0.01	0.01	0.00	0.03	0.02	0.03	0.04	0.00	0.01	0.03
	Puerto Rican	0.07	0.05	**0.12**	0.08	0.06	0.06	0.04	0.05	**0.17**	**0.16**
	Mexican	0.06	0.06	0.05	0.05	0.06	0.05	0.05	0.08	**0.10**	0.07
	White	**−0.11**	−0.08	**−0.11**	**−0.10**	**−0.12**	−0.08	**−0.11**	**−0.12**	**−0.19**	**−0.20**

Bolded numbers are the comparably stronger correlations.

**Table 3:** The top five chemicals contributing to an EPA region’s health risk from industrial air toxins.

EPA REGION 1		EPA REGION 2		EPA REGION 3		EPA REGION 4		EPA REGION 5	
**Chemical**	**Percent Contribution**	**Chemical**	**Percent Contribution**	**Chemical**	**Percent Contribution**	**Chemical**	**Percent Contribution**	**Chemical**	**Percent Contribution**
Chromium	34%	Ethylene oxide	53%	Ethylene oxide	38%	Ethylene oxide	26%	Chromium	27%
Cobalt compounds	18%	Cobalt	12%	Chromium compounds	33%	Chromium	22%	Ethylene oxide	22%
Cobalt	10%	Chromium	10%	Chromium	10%	Chromium compounds	16%	Cobalt	19%
Nickel compounds	9%	Nickel	5%	Nitroglycerin	3%	Arsenic compounds	6%	Chromium compounds	13%
Ethylene oxide	6%	Chromium compounds	4%	Nickel	3%	Cobalt compounds	4%	Cobalt compounds	4%
	77%		83%		87%		74%		86%
**EPA REGION 6**		**EPA REGION 7**		**EPA REGION 8**		**EPA REGION 9**		**EPA REGION 10**	
**Chemical**	**Percent Contribution**	**Chemical**	**Percent Contribution**	**Chemical**	**Percent Contribution**	**Chemical**	**Percent Contribution**	**Chemical**	**Percent Contribution**
Ethylene oxide	59%	Chromium	39%	Ethylene oxide	61%	Chromium	24%	Chromium compounds	49%
Chromium	8%	Ethylene oxide	22%	Chromium	14%	Ethylene oxide	20%	Cobalt	20%
1,3-Butadiene	7%	Chromium compounds	18%	Arsenic compounds	12%	Chromium compounds	17%	Chromium	9%
Chloroprene	5%	Nickel	5%	Chromium compounds	2%	Cobalt	8%	Nickel	7%
Propyleneimine	4%	Nitroglycerin	3%	Hydrogen sulfide	2%	Arsenic compounds	7%	Formaldehyde	5%
	84%		87%		91%		76%		89%

**Table 4:** Results from the linear mixed model predicting tract-level PM_10_ exposure.

Percent Tract:	Model 1			Model 2			Model 3			Model 4			Model 5			Model 6		
	coeff.		SE	coeff.		SE	coeff.		SE	coeff.		SE	coeff.		SE	coeff.		SE
White	ref.			ref.			ref.			ref.			ref.			ref.		
Hispanic	5.48	**	0.08															
African American	2.23	**	0.07	2.42	**	0.07	2.40	**	0.07	2.26	**	0.07	2.25	**	0.07	2.01	**	2.01
Hispanic White				6.83	**	0.11	3.74	**	0.13									
Hispanic Black				−5.19	**	1.55	−7.82	**	1.52									
Foreign-Born							7.74	**	0.15				7.71	**	0.15	6.52	**	0.16
Cuban										9.65	**	0.41	2.84	**	0.42	3.98	**	0.44
Dominican										−7.98	**	0.61	−10.51	**	0.60	−8.23	**	0.61
Mexican										6.35	**	0.10	4.15	**	0.11	4.01	**	0.12
Puerto Rican										2.53	**	0.38	2.63	**	0.37	0.61	**	0.38
Unemp. (White)																−0.28		0.20
Unemp. (African American)																−0.60	**	0.08
Unemp. (Hispanic)																−0.48	**	0.10
Intercept	16.15	**	0.42	16.21	**	0.42	15.88	**	0.42	16.23	**	0.41	15.84	**	0.41	16.35	**	0.41
State (EPA Region)	8.58			8.60			8.51			8.25			8.25			8.27		
Residual	13.47			13.62			13.15			13.44			12.97			12.30		
AIC	391,403.10			392,154.30			389,632.00			391,220.40			388,675.10			304,573.10		
BIC	391,406.90			392,158.10			389,635.80			391,224.20			388,678.90			304,576.80		
N (tracts)	71,905			71,905			71,905			71,905			71,905			71,905		

Note: All models included population density as a control. ** Significant at a *p*-value of <0.01.

**Table 5:** Results from the linear mixed model predicting tract level PM_2.5_ exposure.

Percent Tract:	Model 1			Model 2			Model 3			Model 4			Model 5			Model 6		
	coeff.		SE	coeff.		SE	coeff.		SE	coeff.		SE	coeff.		SE	coeff.		SE
White	ref.			ref.			ref.			ref.			ref.			ref.		
Hispanic	1.69	**	0.02															
African American	1.58	**	0.02	1.64	**	0.02	1.63	**	0.02	1.57	**	0.02	1.56	**	0.02	1.33	**	0.02
Hispanic White				1.97	**	0.03	1.20	**	0.04									
Hispanic Black				−2.16	**	0.44	−2.81	**	0.43									
Foreign-Born							1.92	**	0.04				1.97	**	0.04	1.70	**	0.04
Cuban										0.86	**	0.11	−0.88	**	0.12	−0.80	**	0.12
Dominican										−1.76	**	0.17	−2.41	**	0.17	−1.85	**	0.17
Mexican										2.04	**	0.03	1.48	**	0.03	1.45	**	0.03
Puerto Rican										1.73	**	0.11	1.75	**	0.10	1.61	**	0.11
Unemp. (White)																0.16		0.06
Unemp. (African American)																−0.04	*	0.02
Unemp. (Hispanic)																−0.08	*	0.03
Intercept	6.96	**	0.18	6.99	**	0.18	6.91	**	0.18	6.98	**	0.18	6.88	**	0.18	7.03	**	0.18
State (EPA Region)	1.67			1.64			1.62			1.64			1.63			1.65		
Residual	1.07			1.09			1.06			1.06			1.03			0.9705		
AIC	209,072.70			210,537.00			208,614.40			208,866.60			206,769.90			160,182.00		
BIC	209,076.50			210,540.80			208,618.20			208,870.40			206,773.60			160,185.80		
N (tracts)	71,905			71,905			71,905			71,905			71,905			71,905		

Note: All models included population density as a control. ** Significant at a *p*-value of <0.01. * Significant at a *p*-value of <0.05.
